# High production of recombinant protein using geminivirus-based deconstructed vectors in *Nicotiana benthamiana*

**DOI:** 10.3389/fpls.2024.1407240

**Published:** 2024-07-23

**Authors:** Nan-Sun Kim, Kyeong-Ryeol Lee, Jihyea Lee, Eui-Joon Kil, Juho Lee, Seon-Kyeong Lee

**Affiliations:** ^1^ Department of Agricultural Biotechnology, National Institute of Agricultural Sciences, Rural Development Administration, Jeonju, Republic of Korea; ^2^ Department of Plant Medicals, Andong National University, Andong, Republic of Korea

**Keywords:** viral vector, geminivirus, transient expression, turbo green fluorescence protein, *Nicotiana benthamiana*

## Abstract

We focused on the geminiviral vector systems to develop an efficient vector system for plant biotechnology. Begomoviruses and curtoviruses, which belong to the *Geminiviridae* family, contain an intergenic region (IR) and four genes involved in replication, including replication-associated protein (Rep, C1), transcriptional activator (TrAP, C2), and replication enhancer (REn, C3). Geminiviruses can amplify thousands of copies of viral DNA using plant DNA polymerase and viral replication-related enzymes and accumulate viral proteins at high concentrations. In this study, we optimized geminiviral DNA replicon vectors based on tomato yellow leaf curl virus (TYLCV), honeysuckle yellow vein virus (HYVV), and mild curly top virus (BMCTV) for the rapid, high-yield plant-based production of recombinant proteins. Confirmation of the optimal combination by co-delivery of each replication-related gene and each IR harboring the *Pontellina plumata*-derived turbo green fluorescence protein (tGFP) gene via agroinfiltration in *Nicotiana benthamiana* leaves resulted in efficient replicon amplification and robust protein production within 3 days. Co-expression with the p19 protein of the tomato bush stunt virus, a gene-silencing suppressor, further enhanced tGFP accumulation by stabilizing mRNA. With this system, tGFP protein was produced at 0.7–1.2 mg/g leaf fresh weight, corresponding to 6.9–12.1% in total soluble protein. These results demonstrate the advantages of rapid and high-level production of recombinant proteins using the geminiviral DNA replicon system for transient expression in plants.

## 1 Introduction

Plant-based expression systems have emerged as promising alternatives to traditional systems, such as mammalian cells, insect cell cultures, and yeast or bacterial fermentation. Plant-derived recombinant proteins have many advantages, including high scalability, low upstream costs, and improved safety (Gleba et al., 2014; Tusé et al., 2014; Chen and Davis, 2016). Both stable transformation and transient expression approaches have been investigated for producing recombinant proteins in plants. Although using stable transgenic plants are well-established, *Agrobacterium*-mediated transient expression is an attractive alternative because it significantly reduces development and production timelines. Although plant-based production of recombinant proteins has been greatly improved by the use of transient expression systems, the low yield of some proteins remains a problem, and efforts to increase expression levels (Rybicki, 2009; Regnard et al., 2010; Naseri et al., 2019). Various viral vectors can be used to amplify target genes, leading to the increased expression of recombinant proteins. Previous studies have used plant RNA viruses, such as alfalfa mosaic virus (Yusibov et al., 2002), cowpea mosaic virus (CPMV) (Montague et al., 2011), potato virus X (Mardanova et al., 2017; Sindarovska and Kuchuk, 2021), and tomato mosaic virus (Kobayashi et al., 2024), to create plant expression vectors that can increase the expression of recombinant proteins in plants. In addition, some DNA viruses, such as geminiviruses, have moved to the spotlight as highly effective expression vectors (Hefferon, 2014; Bhattacharjee and Hallan, 2022).

Geminiviruses are a family of plant viruses with circular, single-stranded DNA genomes (Stanley, 1985). These viruses are characterized by a relatively small genome (~3.0 kb) and can replicate their genome in enormously large copy numbers in infected (Timmermans et al., 1994). Although they are highly effective for recombinant protein expression in plants, their cloning capacity is limited, and they have a narrow host range. A deconstructed viral vector approach, which involves the removal of undesired viral genes related to movement and coat proteins and their replacement with an expression cassette, resulting in a smaller and more efficient vector, has been developed to overcome these limitations (Gleba et al., 2004). Replicons replicate after delivery to the plant cells and increase the copy number of the carried DNA, leading to high levels of target gene expression (Lozano-Durán, 2016). This approach has been successfully used to generate geminiviral replicon-based deconstructed vectors for the expression of reporter proteins, antigen proteins as vaccine candidates, and monoclonal antibodies (Huang et al., 2009, 2010; Baltes et al., 2014; Cermák et al., 2015; Wang et al., 2017; Diamos and Mason, 2018a).

Tomato yellow leaf curl virus (TYLCV) and honeysuckle yellow vein virus (HYVV) belong to the genus *Begomovirus* (Navot et al., 1991; Argüello-Astorga et al., 1994; Wang et al., 2011), while beet mild curly top virus (BMCTV) is a member of the genus *Curtovirus* (Soto and Gilbertson, 2003), which have a monopartite genome. Monopartite begomoviruses and curtoviruses have similar genomic structures and encode 6 or 7 multifunctional proteins (Fondong, 2013; Zerbini et al., 2017). They have an intergenic region (IR), including the origin of replication and bidirectional promoters for the expression of viral genes, and encode two or three proteins in the virion-sense strand, V1, V2, and V3 (curtoviruses only), and four proteins in the complementary strand, C1, C2, C3, and C4.

The replication of each T-DNA replicon is seen as being crucial for gene amplification in geminivirus replication systems. For this, it is essential IR and Rep protein encoded by the C1 ORF. IR carries the universal TAATATT/AC motif, which is required for the cleavage and joining of viral DNA during replication (Laufs et al., 1995a, b). The replication-associated protein (Rep) encoded by the C1 open reading frame (ORF) is conserved in sequence, position, and function. Rep is essential for rolling circle replication (RCR) in geminiviruses and initiates DNA replication under the control of the bidirectional core promoter in IR (Fondong, 2013). Rep binds to the Rep complex-binding site, which contains a directly repeated sequence between the TATA box and the transcription start site, to initiate RCR (Fontes et al., 1992) (**Supplementary Figure S1**). Transcription activation protein (TrAP) encoded by the C2 ORF is a multifunctional protein involved in gene activation, viral pathogenicity, and suppression of gene silencing (Pantaleo, 2011; Wang et al., 2012). The replication enhancer (REn), encoded by the C3 ORF, enhances viral DNA accumulation via interaction with the Rep protein and promotes symptom development (Shivaprasad et al., 2005; Pasumarthy et al., 2010; Lozano-Durán, 2016). Taken together, these proteins have evolved into a multifunctional nature and have contributed to the viral genetic economy because of their very small genomes. Protein multifunctionality, together with overlapping genes, is evidence of the plasticity through which evolution brings together functional domains into a single polypeptide chain. Although many of these interactions and their biological functions have been elucidated, the optimization of viral replication systems for the production of recombinant proteins in plant-based transient expression systems remains poorly understood.

*Nicotiana benthamiana* is one of the most widely used hosts for the transient expression of recombinant proteins because of its advantages over other plant systems, including fast growth, high biomass, and efficient infiltration of *A. tumefaciens* (Goodin et al., 2008; Chen and Lai, 2013). These systems with virus-based vectors can produce high-level expression of recombinant proteins, sometimes accumulating more than 5 g of target proteins per kilogram of leaf fresh weight within a few days (Matoba et al., 2011; Diamos and Mason, 2018a; Yamamoto et al., 2018). It is conceivable that *N. benthamiana*-based virus expression systems are attractive and may be of significant importance in plant-based recombinant protein production systems.

Deconstructed viral vectors based on three Geminiviridae-belonging viruses, TYLCV, HYVV, and BMCTV, were constructed for high expression of recombinant proteins in this study. We used *Pontellina plumata*-derived turbo GFP (tGFP) with each IR and co-delivered it to each replication-related gene (C1/C2/C3) for transient expression in *N. benthamiana* leaves. We cloned the C1 (C1 and modified C2), C12 (C1, C2, and modified C3), or C123 (C1, C2, and C3) genes of the three geminiviruses and fused the cauliflower mosaic virus (CaMV) 35S short promoter (s35SP) for driving expression, and each IR-carrying tGFP under the control of the 35S double promoter (d35SP) and PinII terminator (PinIIT) in a plant expression vector. The rapidity, simplicity, and high yield potential of this vector system greatly enhance the commercial feasibility of recombinant protein production in plants.

## 2 Materials and methods

### 2.1 Cloning of geminiviral DNA elements and vector construction

The MoClo system, based on Golden Gate cloning technology, has been used for viral gene cloning and binary vector construction for plant transformation (Weber et al., 2011; Engler et al., 2014; Marillonnet and Grützner, 2020). Briefly, restriction-ligations were set up in one tube containing approximately 20 fmol of each plasmid DNA, 10 U of the required type IIS restriction enzymes, such as *Bsa*I and *Bpi*I (New England BioLabs, Frankfurt, Germany), 10 U T4 DNA ligase, and 1×T4 DNA ligase buffer (Fermentas, St. Leon-Rot, Germany) in a final reaction volume of 20 μL. The reaction was incubated in a thermocycler for 5 hours at 37°C, 5 min at 50°C and 10 min at 80°C. The mixture was transformed into competent DH5α cells using heat shock. Colonies were selected on LB plates containing appropriate antibiotics and extrapolated for the entire transformation.

Level 0 (L0) fragments were synthesized as linear fragments and cloned into the pBHK cloning vector (Bioneer, Daejeon, Republic of Korea). Adaptor sequences were introduced at both the 5′- and 3′- ends of the recognition site of *Bpi*I or *Bsa*I type IIS restriction enzyme as design changes prior to synthesis during initial assembly. For geminiviral-based deconstructed vector construction, viral sequences for L0 parts were based on the genome of the TYLCV Korean Isolate (2,774 bp, GenBank accession no. KF225312), the HYVV Korean Isolate (2,763 bp, GenBank accession no. GQ477135), and the BMCTV Worland isolate (2,930 bp, GenBank accession no. U56975). Overlapping complementary sense ORFs and the C1, C2, and C3 genes were modified and synthesized. For the replication-related gene expression vectors, the C3 start codon harbored in C12 (C1 and C2), and the C2 start codon harbored in C1 were modified to prevent spontaneous C3 and C2 truncation, respectively. The complete sequences are shown in **Supplementary Figures S2**-**S5**. ORFs of each synthesized replication-related genes in pBHK (L0), s35SP (L0; pICH41388) and NosT (L0; pICH41421) were inserted into Level 2 destination vector (pICH86969) using Golden Gate cloning method with *Bsa*I to make binary vectors as follows: TYLCV-based replication-related gene vectors, pTC1, pTC12, pTC123, HYVV-based replication-related gene vectors, pHC1, pHC12, pHC123, and BMCTV-based replication-related gene vectors, pBC1, pBC12, and pBC123, respectively.

**Figure 1:**
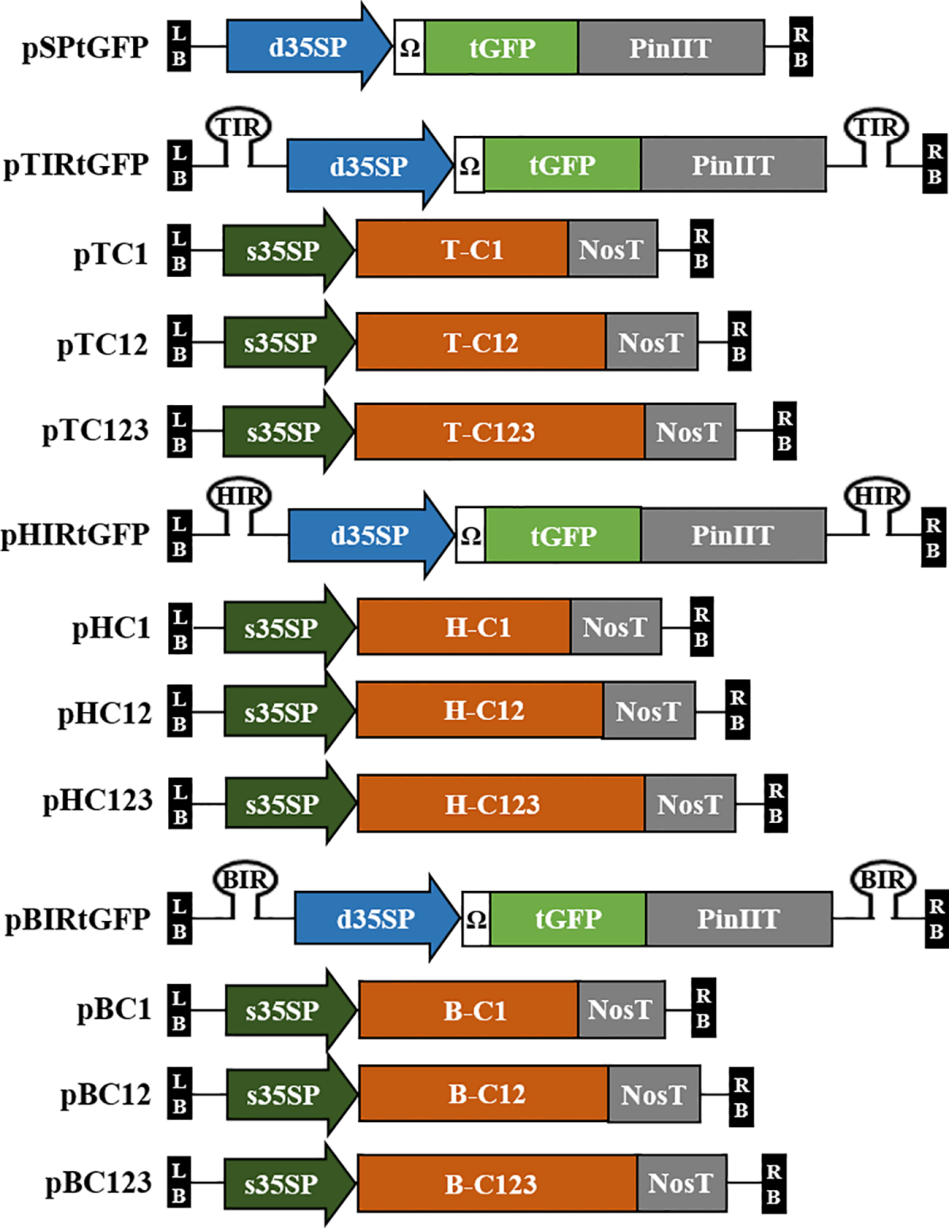
Diagrams of the T-DNA region of the geminivirus-based deconstructed vectors used in this study. Plasmid pSPtGFP contains *tGFP* under the CaMV 35S double promoter (d35SP) with 5′-leader sequence of tobacco mosaic virus (Ω) and potato protease inhibitor II terminator (PinIIT). The intergenic region (IR) of TYLCV, HYVV, or BMCTV is located in pSPtGFP inside of T-DNA with a hairpin structure cloned into the Level M vector and named pTIRtGFP, pHIRtGFP, and pBIRtGFP. C1 (C1/ΔC2; start codon of C2 was modified), C12 (C1/C2/ΔC3; start codon of C3 was modified) and C123 (C1/C2/C3) of TYLCV, HYVV, or BMCTV ORFs that encode for replication initiation protein were cloned into Level 2 vector under CaMV 35S short promoter (s35SP) and Nos terminator (NosT). The vectors were named pTC1, pTC12, and pTC123 with TYLCV ORFs; pHC1, pHC12, and pHC123 with HYVV ORFs; pBC1, pBC12, and pBC123 with BMCTV ORFs, respectively. LB and RB indicate the T-DNA left and right border, respectively.

Plant codon-optimized *Pontellina plumata*-derived turbo GFP (tGFP, level 0; pICSL80005) was used as a reporter gene in this study. tGFP (L0; pICSL80005), d35SP, tobacco mosaic virus (TMV) 5′-leader sequence (Ω) (L0; pICH51288), and PinIIT (L0; pBHK) were inserted into Level 1-2 destination vector (L1-2; pICH47751) using Golden Gate cloning method with *Bsa*I to construct expression vector pSPtGFP, i.e., the GFP expression vector. Each IR was cloned into L1-1 and L1-3 using PCR and specific primers (**Supplementary Table S1**). Each IR (in L1-1 and L1-3), pSPtGFP (L1-2), and end linker L3E were inserted into the Level M1 destination vector (pAGM8031) using the Golden Gate cloning method with *Bpi*I to construct IR-carrying *tGFP* expression vectors, i.e., TYLCV-based IR vector, pTIRtGFP; HYVV-based IR vector, pHIRtGFP; and BMCTV-based IR vector, pBIRtGFP. The pSPtGFP expression vector served as a nonreplicating control. A schematic diagram of the geminivirus-based deconstructed vectors is shown in **Figure 1**.

### 2.2 *Agrobacterium tumefaciens*-mediated transient expression

Binary vectors were separately introduced into *A. tumefaciens* GV3101 by using 50–500 ng of recombinant plasmid in the freeze-thaw method, as described by Jyothishwaran et al. (2007). Recombinant strains were grown overnight at 28°C with agitation in LB medium supplemented with appropriate antibiotics for infiltration. Cells were pelleted using centrifugation 2,000 × *g* for 5 min, and then suspended in infiltration buffer [10 mM 2-(*N*-morpholino) ethanesulfonic acid (MES) (pH 5.6) and 10 mM MgCl_2_ supplemented with 100 μM acetosyringone] and diluted in infiltration buffer to OD_600_ = 0.5, unless otherwise described. When mixing the two constructs, each *Agrobacterium* concentration was set to OD_600_ = 1.0, and mixed 1:1. For co-infiltration with tomato bushy stunt virus (TBSV) p19 (kindly provided by Prof. Inhwan Hwang, POSTECH, Republic of Korea), each was set to OD_600_ = 1.0, and mixed 1:1:0.5. After incubation at 25°C for 3 h, cells were infiltrated into the leaves of 4–5 weeks old *N. benthamiana* plants grown in a hydroponic growing media at 23°C under a 16 h light and 8 h dark photoperiod at a light intensity of 100 mol m^-2^ s^-1^. The resulting bacterial suspensions were infiltrated using syringe without needles into fully expanded leaves through a small puncture (Huang et al., 2006).

### 2.3 Polymerase chain reaction analysis

Genomic DNA was isolated from plant tissue using the NucleoSpin Plant II (Machery-Nagel GmbH & Co, Düren, Germany) as per the manufacturer’s instructions. Genomic DNA was quantified on a NanoDrop spectrophotometer (Thermo Scientific, Waltham, MA, USA) and diluted to 50 ng/μL. PCR was performed in reaction volumes of 20 μL using 100 ng of genomic DNA with Ex-Taq DNA polymerase (TaKaRa, Shiga, Japan). The primers (**Supplementary Table S1**) used in the PCR analysis were designed to amplify each replicon recirculation and C1, C12, and C123 in the co-infiltrated *N. benthamiana* genome. The PCR products were electrophoresed on 1.0% (w/v) agarose gels, stained with StaySafe Nucleic Acid Gel Stain (Real Biotech Corporation, Taipei, Taiwan), and visualized under ultraviolet light.

Quantitative PCR was performed using a CFX96 Touch Real-Time PCR Detection System (Bio-Rad, Hercules, CA, USA) real-time PCR machine together with AccuPower 2×GreenStar qPCR Master Mix (Bioneer). Triplicate reactions were performed, and *tGFP* gene copy numbers were normalized using β-actin of *N. benthamiana* (GenBank accession no. JQ256516) as the reference. PCR primer sequences are listed in **Supplementary Table S1**.

### 2.4 Quantitative real-time PCR

Total RNAs was purified from infiltrated *N. benthamiana* leaves at 1, 3, 5, and 7 day post infiltration (DPI) using the Spectrum™ Plant Total RNA Kit (Sigma-Aldrich, St. Louis, MO, USA), and the residual DNA was removed with RNase-free DNase I (Takara, Shiga, Japan). First-strand cDNA was synthesized from 5 μg of total RNA and oligo dT primers using RNA-to-cDNA EcoDry Premix (Takara, Shiga, Japan) according to the manufacturer’s protocol. Quantitative real-time PCR analysis was performed with 100 ng of cDNA in a 20 μL reaction volume using AccuPower 2×GreenStar™ qPCR Master Mix (Bioneer, Daejeon, Republic of Koread). Gene-specific primers used are listed in **Supplementary Table S1**. qRT-PCR was performed with an initial step at 95°C for 10 min followed by 40 cycles of 95°C for 20 s, 58°C for 20 s, and 72°C for 25 s. Fluorescence was recorded after the last step of every cycle. Three replicates were performed for each sample. Amplification, data processing, and detection were performed using the CFX96 Real-Time PCR Detection System (Bio-Rad, Hercules, CA, USA). Quantification cycle (Cq) values were examined using the 2^-ΔCT^ method to determine changes in gene expression.

### 2.5 Protein extraction

Total soluble proteins were extracted by homogenizing agroinfiltrated leaf samples harvested 1, 3, 5, and 7 DPI with 1:2 (w/v) ice-cold extraction buffer [200 mM Tris-Cl (pH 7.0), 100 mM NaCl, 10 mM EDTA, 0.5% Triton X-100, and a protease inhibitor cocktail (Roche Diagnostics GmbH, Mannheim, Germany)]. Cleared supernatants were obtained by centrifugation at 15,000 × *g* for 15 min at 4°C. The protein concentration in the leaf samples was determined using the Bradford Protein Assay Reagent kit (Bio-Rad, Hercules, CA, USA) (Bradford, 1976) with bovine serum albumin as the reference standard.

### 2.6 Turbo GFP fluorescence assay

The tGFP samples were prepared by dilution in extraction buffer and 100 μL of each sample was added to a 96-well plate (SPL, Republic of Korea) in duplicate. The tGFP fluorescence intensity was examined using a Victor^3^ microplate reader (PerkinElmer, Waltham, MA, USA). The excitation and emission wavelengths were 485 nm and 538 nm, respectively. All measurements were performed at 25°C and the negative control (extract of un-infiltrated plant leaves) was subtracted before graphing. *E. coli*-derived tGFP protein (Evrogen, Moscow, Russia) was used to generate a standard curve. Leaves expressing tGFP harvested at 1, 3, 5, and 7 DPI were viewed under UV illumination generated by a LAS4000 (Fujifilm, Tokyo, Japan).

### 2.7 Western blot analysis

Total soluble protein (25 μg) was mixed with sample buffer [10% glycerol, 60 mM Tris–HCl (pH 6.8), 2% SDS, 0.5M dithiothreitol, 0.01% bromophenol blue] at 25°C and were separated using 12% SDS-polyacrylamide gel electrophoresis and then electrophoretically transferred to a iBlot 2 polyvinylidene fluoride (PVDF) Regular Stacks (Invitrogen, Carlsbad, CA, USA) following the manufacturer’s instructions. The protein-transferred membranes were blocked with 5% non-fat skim milk in Tris-buffered saline containing 0.05% Tween-20 (TBST, pH 7.4) for 2 h at 25°C. tGFP protein was detected with mouse anti-tGFP monoclonal antibody (Origene, Rockville, MD, USA) at 1:5,000 dilutions. The secondary antibody used was alkaline phosphatase-conjugated goat anti-mouse IgG (Sigma-Aldrich, St. Louis, MO, USA) at 1:10,000 dilutions. The membranes were developed using nitro blue tetrazolium chloride and 5-bromo-4-chloro-3-indolyl phosphate (Sigma-Aldrich, St. Louis, MO, USA). Purified *E. coli* expressing tGFP protein (Evrogen, Moscow, Russia) was used as a positive control.

### 2.8 Enzyme-linked immunosorbent assay

The amount of tGFP protein extracted from the agroinfiltrated leaf tissues was determined using indirect ELISA. Briefly, a 96-well Maxisorp microtiter plate (NUNC, Roskilde, Sjelland, Denmark) was coated with tGFP plant extracts in coating buffer (15 mM Na_2_CO_3_, 35 mM NaHCO_3_, pH 9.6) at 4°C overnight, and washed with 200 μL of PBST (PBS with 0.1% Tween-20, pH 7.4) four times. The wells were then blocked with 1% BSA in PBS at 37°C for 2 h. After washing the wells three times, 100 μL of diluted anti-mouse tGFP monoclonal antibody (1:5,000) (Origene, Rockville, MD, USA) were added to the wells and incubated at 37°C for 2 h. After washing four times, anti-mouse IgG-conjugated horseradish peroxidase (1:10,000) (GenDepot, Baker, TX, USA) was added to each well and incubated at 37°C for 2 h. After five times washing, 100 μL of TMB (3,3′,5,5′-tetramethylbenzidine) containing H_2_O_2_ solution were added and the plate was incubated for 15 min at 25°C. The enzyme reaction was stopped by quickly pipetting 50 μL of 2 N H_3_PO_4_ into each well. The absorbance at 450 nm was measured using a microplate reader. To calculate the relative amount of tGFP in the plant sample, the OD value from each sample was subtracted from the untransformed plant OD value before converting by reference to an ELISA standard curve constructed with purified bacterial tGFP (Evrogen, Moscow, Russia). Plant samples were analyzed by diluting from 1:1,000 to 1:4,000 with a coating buffer.

## 3 Results

### 3.1 Geminivirus-based deconstructed viral vector construction

We deconstructed TYLCV, HYVV, and BMCTV strains belonging to the *Geminiviridae* family to develop viral vector systems for the high expression of target proteins. These vectors were based on the replication machinery related to IR and Rep to induce viral replicons and the transcription unit of the target recombinant protein gene instead of the sequence-coding coat and movement proteins (Gleba et al., 2004). This system used two vectors containing different portions of the TYLCV, HYVV, or BMCTV genomes. The first vector carried the transcription unit of *tGFP* gene and the IRs of the TYLCV, HYVV, or BMCTV genome, which are essential for each episomal replication, incorporating at its 5′- and 3′-ends, and the second vector contained each replication-related gene.

We modified and constructed vectors using MoClo systems to investigate the efficient combination of each IR and replication-related gene for the amplification of episomal replicons and high protein expression. Each IR-carrying tGFP vector, i.e., pTIRtGFP, pHIRtGFP, and pBIRtGFP, was controlled by d35SP, Ω, and PinIIT, respectively. PinIIT is a strong terminator (An et al., 1989) and reports have shown that the use of PinIIT results in 10–50 times greater HBsAg accumulation and 8.5−fold higher GFP fluorescence than NosT (Richter et al., 2000; Diamos and Mason, 2018b). pSPtGFP was used as the negative control (**Figure 1**). Each Rep-supplying vector, i.e., C1ΔC2 (named pTC1, pHC1, and pBC1), C1/C2/ΔC3 (named pTC12, pHC12, and pBC12), and C1/C2/C3 (named pTC123, pHC123, and pBC123), was controlled by s35SP and NosT. *Agrobacterium* strains carrying these constructs were co-infiltrated at the same ratio, and *N. benthamiana* leaves were then sampled in a time-dependent manner.

### 3.2 Amplification of episomal replicons induced an increase in the *tGFP* gene copies

**Figure 2:**
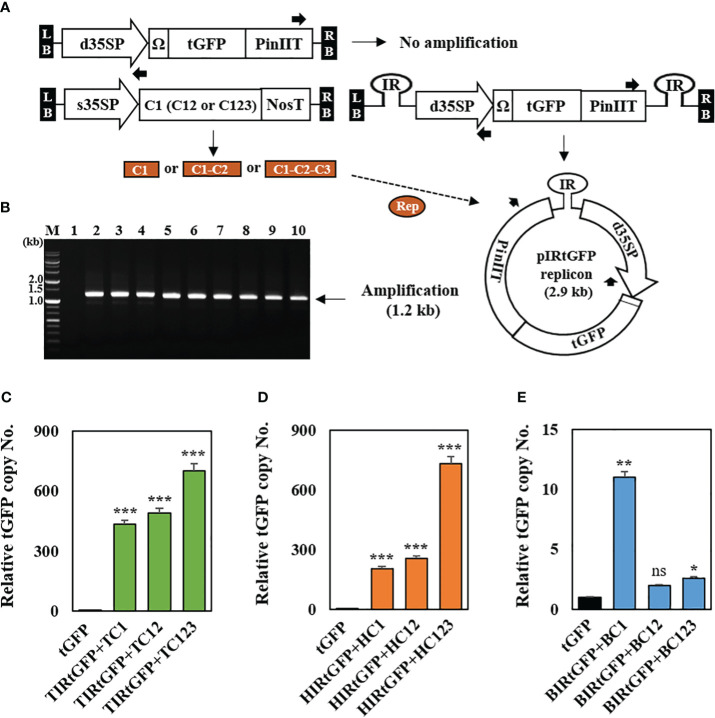
Conformation of IR-carrying *tGFP* replicons in co-infiltrated *N. benthamiana* leaves with different vector combinations. **(A)** Outward-facing primer design, which can be only amplified using replicon DNA as a template. **(B)** Genomic PCR products using outward-facing primers. Lane M: 1 kb DNA ladder, Lane 1: tGFP only, Lanes 2–4: co-infiltrated leaf with TIRtGFP+TC1, TC12 or TC123, Lanes 5–7: co-infiltrated leaf with HIRtGFP+HC1, HC12 or HC123, Lanes 8–10: co-infiltrated leaf with BIRtGFP+BC1, BC12 or BC123. (C to E) The transgene copy number analyzed via qPCR with primers specific for tGFP in Agroinfiltrated *N. benthamiana* leaves. **(C)** The *tGFP* copy number of co-infiltration with TIRtGFP+TC1, TC12, or TC123. **(D)** The *tGFP* copy number of co-infiltration with HIRtGFP+HC1, HC12, or HC123. **(E)** The *tGFP* copy number of co-infiltration with BIRtGFP+BC1, BC12, or BC123. tGFP in a single agroinfiltrated leaf was used as a negative control. Relative copy number was normalized using *tGFP* expression at 3 DPI. Black arrows represent primer binding sites for the polymerase chain reaction. Data means ± SE from three independent infiltrated samples. Significant differences were assessed via Dunnett’s one-way ANOVA. *p < 0.05; **p < 0.01; ***p < 0.001; ns, not significant.

Following a previous report, genomic DNA was extracted from 3 DPI leaf samples (Regnard et al., 2010) to confirm the formation of episomal replicons containing three different IRs, respectively, in co-infiltrated leaves. The formation of episomal replicon from T-DNA is essential for the amplification using the primers oriented for both ends (**Figure 2A**). Approximately 1.2 kb PCR products were amplified an only from circularized unit-length replicons (**Figure 2B**). The results showed that replicon formation occurred in the co-infiltrated *N. benthamiana* leaves. We also tested whether the amplification of episomal DNA was associated with an increase in the *tGFP* copy number. Real-time PCR was used to determine the *tGFP* gene copy number in co-infiltrated *N. benthamiana* leaves with each replicon vector combination. In combination with TIRtGFP and replication-related genes, *tGFP* gene amplification levels were high, ranging from 433- to 701-times compared to the negative control (**Figure 2C**). In addition, in combination with HIRtGFP and replication-related genes, *tGFP* gene amplification levels increased 201- to 731-times compared with the negative control (**Figure 2D**). The data indicated that the highest tGFP amplification was achieved with the combination of TIRtGFP+TC123 or HIRtGFP+HC123, which was 701- to 731-times increase compared to that in the negative control. However, in the case of the combination of BIRtGFP and replication-related genes, the *tGFP* gene amplification level showed a 2- to 11-times increase compared to the negative control (**Figure 2E**). Co-infiltration with BIRtGFP+BC1 resulted in the highest copy number (11-times) of *tGFP* gene among the combinations of BIRtGFP and BMCTV replication-related genes, which was different from the other cases (**Figures 2C–E**).

### 3.3 Episomal replication resulted in enhanced *tGFP* mRNA expression and fluorescence intensity

The impact of *tGFP* gene amplification on transient expression was determined by comparing *tGFP* expression of the three geminivirus-based deconstructed vectors. We investigated the potential of various geminivirus-derived IR- and replication-related gene combinations to enhance *tGFP* mRNA expression and fluorescence. Plant extracts prepared from samples taken from agroinfiltrated *N. benthamiana* leaves over 7 d were analyzed using qRT-PCR and tGFP fluorescence.

**Figure 3:**
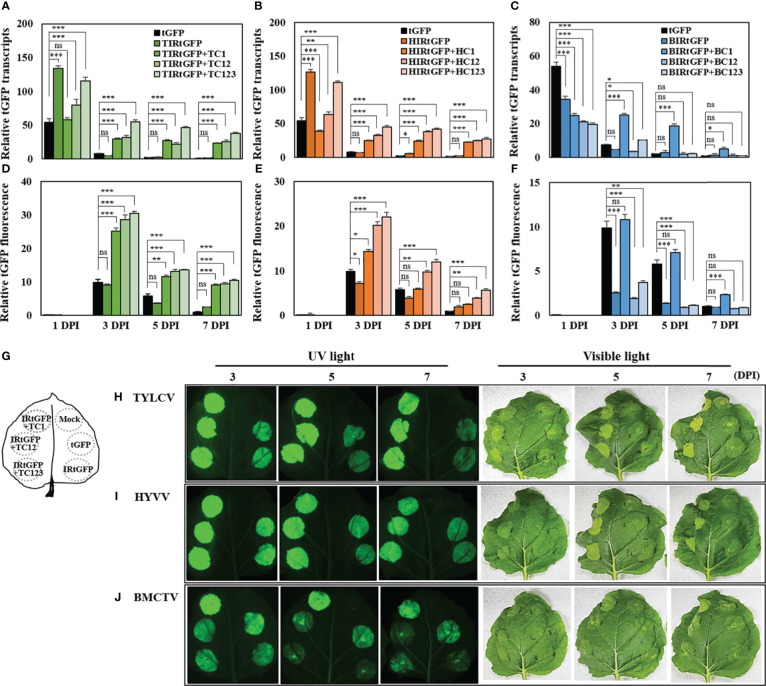
mRNA expression and fluorescence analysis of *tGFP* in co-infiltrated *N. benthamiana* leaves with different vector combinations over times. Total RNA and total soluble proteins were extracted from agroinfiltrated leaf tissues. **(A−C)** mRNA of *tGFP* analyzed via qRT-PCR with specific primers for tGFP and **(D−J)** fluorescence assay analyzed via microplate reader. **(A, D)** Co-infiltration with TIRtGFP and TC1 or TC12 or TC123 and TIRtGFP; **(B, E)** Co-infiltration with HIRtGFP and HC1 or HC12 or HC123; **(C, F)** Co-infiltration with BIRtGFP and BC1 or BC12 or BC123 in *N. benthamiana*. Relative transcripts and fluorescence of tGFP were normalized to tGFP single agroinfiltrated leaf at 7 DPI. tGFP is a single agoinfiltrated leaf as a negative control. **(G)** Illustration of the approach to directly compare *tGFP* expression between different vector combinations. Images are representative tGFP fluorescence intensity and photograph of infiltrated leaves in **(H)** TYLCV, **(I)** HYVV, and **(J)** BMCTV at 3, 5, and 7 DPI under UV and visible light. Data means ± SE from three independent infiltrated samples. Significant differences were assessed via Dunnett’s one-way ANOVA. *p < 0.05; **p < 0.01; ***p < 0.001; ns, not significant.

The relative mRNA expression of *tGFP* in each vector combination peaked at 1 DPI, after which there was a steady decline. The mRNA expression patterns at 3 DPI varied depending on the presence or absence of geminivirus-derived replication-related factors. In the case of *tGFP* only, the relative mRNA expression decreased to 87% at 3 DPI, and IR-carrying *tGFP* only, TIRtGFP, HIRtGFP, and BIRtGFP also sharply declined to 96.3%, 94.5%, and 88.2%, respectively, at 3 DPI (**Figures 3A–C**). However, when co-expressed with each IR and replication-related gene, relative mRNA expression was maintained at 47.8, 38.8, and 50% in TIRtGFP+TC123, TC12, or TC1 (**Figure 3A**) and 40.5, 51.6, and 63.1% in HIRtGFP+HC123, HC12, and HC1 (**Figure 3B**), respectively, compared to that of the negative control at 3 DPI. In contrast, BMCTV-related combinations showed lower mRNA expression in all combinations compared to the negative control at 1 DPI and dropped quickly as in the controls, with the exception of BIRtGFP+BC1, which was maintained at 99.8% at 3 DPI (**Figure 3C**).

The expression levels among vector combinations were further compared by determining the fluorescence intensity of the crude extract of agroinfiltrated *N. benthamiana* leaf tissues transiently expressing *tGFP* with various vector combinations. Unlike the maximum mRNA expression at 1 DPI, no tGFP fluorescence was detected, and the highest fluorescence was quantified at 3 DPI for all combinations, after which it decreased. Leaf tissues co-infiltrated with TIRtGFP+TC123, HIRtGFP+HC123, or BIRtGFP+BC1 showed the highest fluorescence intensity of vector combinations with high mRNA expression (**Figure 3**). The relative fluorescence intensities of TIRtGFP+TC123, HIRtGFP+HC123, and BIRtGFP+BC1 at 3 DPI increased 31-, 22-, and 11-fold, respectively, compared to the negative control on 7 DPI (**Figures 3D–F**). When *tGFP* was expressed from TIRtGFP+TC123 at 3 DPI, higher mRNA expression and fluorescence intensity were observed compared to those of the other combinations and other DPIs (**Figures 3A, D**). These results demonstrated a similar correlation between replicon amplification and *tGFP* expression in various vector combinations, strongly suggesting that higher gene expression is due to the high copy number of replicons.

Each single vector or vector combination was infiltrated into a single leaf to determine the fluorescence intensity and phenotypic changes in tGFP in the infiltrated *N. benthamiana* leaf tissues. An illustration of the approach used to directly compare *tGFP* expression between different vector combinations are presented in **Figure 3G**. Images are representative of tGFP fluorescence intensity and photographs of infiltrated leaves in TYLCV, HYVV, and BMCTV at 3, 5, and 7 DPI under UV and visible light (**Figures 3H–J**). The green fluorescence of the infiltrated areas at 3–7 DPI was brighter than that of the negative control under UV light (**Figures 3H–J**). Severe tissue necrosis was not observed under visible light in agroinfiltrated *N. benthamiana* leaves expressing *tGFP* with a combination of various geminivirus-derived IR and replication-related genes; however, inspection of leaves under visible light revealed that fluorescing leaf regions infiltrated with vector combinations were gradually brighter and curlier than those of the negative control as DPI increased (**Figures 3H–J**).

### 3.4 Post-transcriptional gene silencing suppressor p19 enhanced accumulation of *tGFP* mRNA and protein

**Figure 4:**
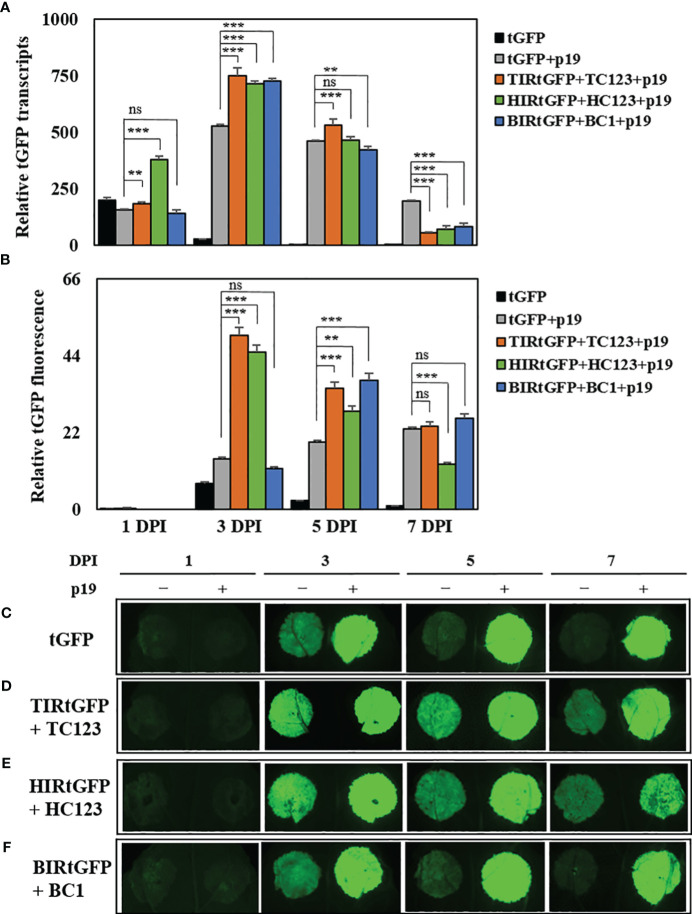
Effects of p19 on *tGFP* transcript level and fluorescence in co-infiltrated *N. benthamiana* leaves. Expression of *tGFP* mRNA in co-infiltrated *N. benthamiana* leaves with TIRtGFP+TC123, HIRtGFP+HC123, and BIRtGFP+BC1 with p19 over time. **(A)** Relative mRNA expression and **(B)** fluorescence assay were normalized using tGFP single agroinfiltrated leaf at 7 DPI. The images are representative of tGFP fluorescence intensity on 1, 3, 5, and 7 DPI through co-expression with p19 in the **(C)** tGFP, **(D)** TIRtGFP+TC123, **(E)** HIRtGFP+HC123, and **(F)** BIRtGFP+BC1 under UV light. Data means ± SE from three independent infiltrated samples. Significant differences were assessed via Dunnett’s one-way ANOVA. **p < 0.01; ***p < 0.001; ns, not significant.

We chose three deconstructed geminiviral vector combinations (TIRtGFP+TC123, HIRtGFP+HC123, and BIRtGFP+BC1) for high production of tGFP. Bottlenecks in gene silencing exist during the transcription in transient expression systems. TBSV p19 co-infiltrated to enhance transcript levels and protein accumulation in *N. benthamiana* leaf tissues by suppressing gene silencing (Qiu et al., 2002). We co-infiltrated a p19 vector with the three most effective combinations based on our data to test whether the expression of p19 in our replicon system can further elevate *tGFP* mRNA levels and fluorescence intensity. We found that co-expression with p19 resulted in a shift in maximal mRNA expression from 1 to 3 DPI. The highest *tGFP* relative mRNA expression reached 529-, 749-, 714-, and 726-times (increased 19.6-, 27.7-, 26.4-, and 26.9-times compared to that of tGFP at 3 DPI, respectively) in the tGFP+p19, TIRtGFP+TC123+p19, HIRtGFP+HC123+p19, and BIRtGFP+BC1+p19 combinations compared to the negative control at 7 DPI, respectively, and then gradually decreased over time in all three combinations (**Figure 4A**).

The fluorescence intensity in tGFP+p19, TIRtGFP+TC123+p19, and HIRtGFP+HC123+p19 reached the highest levels (2-, 7.1-, and 6.4-times, respectively) at 3 DPI, whereas that of BIRtGFP+BC1+p19 reached the highest level (18.5-times) at 5 DPI compared to the negative control of each DPI (**Figure 4B**). It gradually decreased, as did the mRNA expression for all combinations except tGFP+p19, which increased over time (**Figure 4B**). The fluorescence intensity under UV light was much stronger in the leaf spots infiltrated with p19 than in the leaf spots infiltrated without p19 (**Figures 4C–F**).

### 3.5 Geminivirus-based deconstructed vectors can be used in scaling up production of recombinant proteins

**Figure 5:**
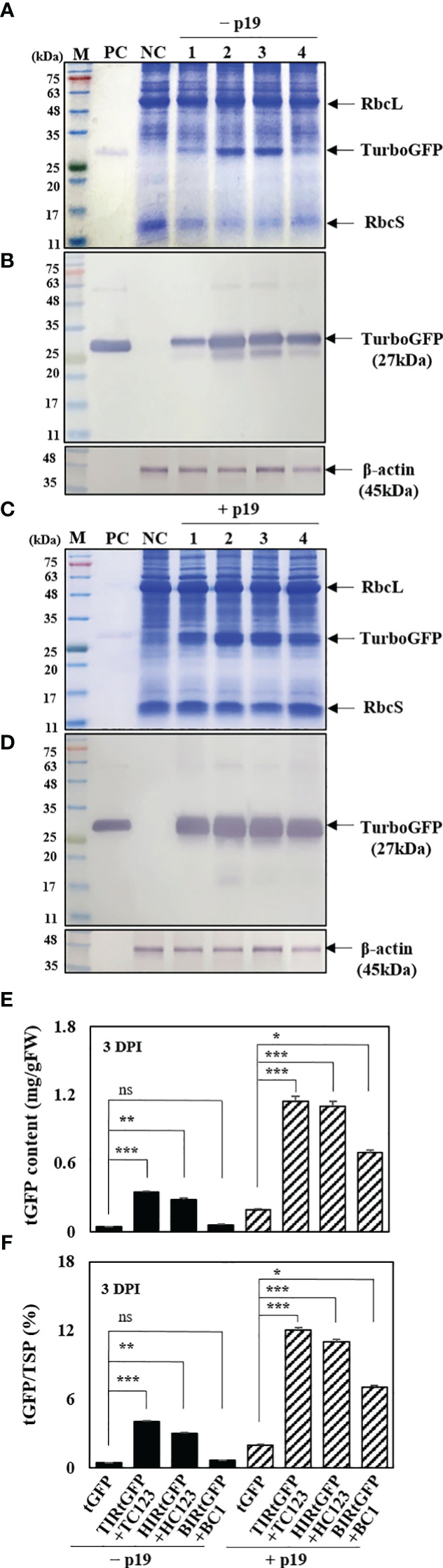
Effects on p19 of tGFP protein expression in co-infiltrated *N. benthamiana* leaves. **(A, C)** SDS-PAGE gels, **(B, D)** Western blot analysis, and **(E, F)** indirect ELISA showing tGFP protein in co-infiltrated *N. benthamiana* leaves both without p19 and with p19 at 3 DPI. Lane M: prestained protein marker; Lane PC: purified bacterial tGFP protein; Lane NC: infiltration buffer as a negative control; Lane 1: infiltration with *tGFP* alone; Lane 2: co-infiltration with TIRtGFP+TC123; Lane 3: co-infiltration with HIRtGFP+HC123; Lane 4: co-infiltration with BIRtGFP+BC1. β-actin used as an internal loading control. Data means ± SE from three independent infiltrated samples. Significant differences were assessed via Dunnett’s one-way ANOVA. *p < 0.05; **p < 0.01; ***p < 0.001; ns, not significant.

The impact of transient protein production was determined using SDS-PAGE, western blot analysis, and enzyme-linked immunosorbent assay (ELISA). Plant extracts were prepared from agro-infiltrated *N. benthamiana* leaves at 3 DPI for determining tGFP protein production. The *E. coli*-derived tGFP as a positive control was observed at 27 kDa and β-actin was used as an internal loading control. In total, 25 μg of leaf total soluble protein (TSPs) from *N. benthamiana* leaf tissue samples at 3 DPI were separated using SDS-PAGE and stained with Coomassie Brilliant Blue. As expected, SDS-PAGE gels showed the three major protein bands in the mass range of 14–45 kDa, i.e., ribulose 1,5-bisphosphate carboxylase oxygenase small (rbcS) and large (rbcL) subunits (Ellis, 1979). In addition, a 27 kDa recombinant tGFP protein were weakly and strongly expressed in agroinfiltrated *N. benthamiana* leaves in all three geminiviral vector combinations without p19 and with p19, respectively (**Figures 5A, C**). A faint 27 kDa tGFP band was detected in tGFP-only and HIRtGFP+HC123 combination, and distinct in TIRtGFP+TC123 combination. However, this was not observed in the BIRtGFP+BC1 combination (**Figure 5A**). In the case of p19 co-expression, TIRtGFP+TC123 and HIRtGFP+HC123 showed stronger tGFP bands compared to that of tGFP-only and BIRtGFP+BC1 (**Figure 5C**). Western blot analysis using a mouse monoclonal tGFP antibody at 3 DPI revealed that *tGFP* expression with p19 was higher than that without p19 in the tGFP-only, TIRtGFP+TC123, HIRtGFP+HC123, and BIRtGFP+BC1 combinations under reducing conditions (**Figures 5B, D**). The production of tGFP in co-infiltrated *N. benthamiana* leaves was estimated using ELISA (**Figure 5E**). When co-expressed with p19, tGFP protein production increased 4.8-, 3.4-, 3.9-, and 11.5-times compared to tGFP alone, TIRtGFP+TC123, HIRtGFP+HC123, and BIRtGFP+BC1 combinations, respectively, reaching the highest levels of 0.1, 1.1, 1.0 and 0.7 mg/g fresh weight (FW), corresponding to 1.9, 12.1, 10.6, and 6.9% TSP, respectively (**Figures 5E, F**). These results indicated that p19 can increase *tGFP* mRNA and protein accumulation, most likely by suppressing post-transcriptional silencing of the transgene in agroinfiltrated *N. benthamiana* leaves.

## 4 Discussion

We constructed the DNA-replicating geminivirus-based deconstructed vectors to enhance the expression of recombinant proteins. It was previously reported that several geminiviral vectors based on mastreviruses [bean yellow dwarf virus (BeYDV), tobacco yellow dwarf virus (TYDV)], and curtovirus [beet curly top virus (BCTV)] for overexpression (Mor et al., 2003; Zhang and Mason, 2006; Kim et al., 2007; Huang et al., 2009; Chung et al., 2011; Dugdale et al., 2013). Several geminivirus-based vectors also have been developed for genome editing and virus-induced gene silencing (Lozano-Durán, 2016; Bhattacharjee and Hallan, 2022; Mahmood et al., 2023). Only TYLCV-based vectors has been reported to enhance protein expression; however, the vector was a disarmed TYLCV viral vector, not a binary vector using an *Agrobacterium*-mediated transformation or transient expression (Peretz et al., 2007).

Viral vectors were developed using IR elements and replication-related genes from TYLCV and HYVV, both of the *Begomovirus* genus of the *Geminiviridae* family, and BMCTV, which belongs to the *Curtovirus* genus. The degree of expression enhancement was examined using tGFP. The findings of this study are as follows: (1) The use of viral vectors led to a significant increase in both the copy number and transcript level of tGFP compared to non-viral vectors. (2) The increase in *tGFP* expression was the highest for TYLCV-based viral vectors, comparable to HYVV-based viral vectors, but significantly lower for BMCTV-based viral vectors. (3) The replication-related genes that most enhanced *tGFP* expression among vectors of the same viral origin were C123 in TYLCV- and HYVV-based vectors and C1 in BMCTV-based vectors. (4) When co-expressed with the silencing suppressor p19, the yield of tGFP co-expressed with HYVV- and TYLCV-derived C123 was approximately 1.1–1.2 mg/g FW and 10.6–12.1% TSP, respectively, indicating a high level of expression.

Viral vectors, including tobamoviruses, potexviruses, tobraviruses, geminiviruses, and comovirus-based vectors, have been developed to enhance the expression of recombinant proteins (Peyret and Lomonossoff, 2015). Among these, tobamovirus-, geminivirus-, and comovirus-based vectors have been the most successful. Using the magnICON system, a hybrid vector composed of elements from TMV, which belongs to the tobamovirus, and turnip vein-clearing virus, has been used to generate very high yields of recombinant proteins, for example, 5 mg/g FW GFP (Marillonnet et al., 2004), 2 mg/g FW plague antigen (Santi et al., 2006), 2.4 mg/g FW hepatitis B virus core antigen (HBcAg) virus-like particles (VLPs) (Huang et al., 2006), and 0.8 mg/g FW Norwalk virus VLPs (Santi et al., 2008). In addition, viral vectors based on CPMV and bean pod mottle virus, both members of the *Comoviridae* with bipartite single-stranded RNA genomes, have been developed (Liu et al., 2005; Sainsbury et al., 2008; Zhang et al., 2010). The CPMV-HT 5′-UTR, which was made hyper translatable by removing the two internal ATG sequences, was inserted between the target gene and the promoter, and this insertion significantly enhanced protein expression, resulting in 1.6 g/kg FW GFP and 1 g/kg FW HBcAg (Sainsbury and Lomonossoff, 2008). This led to the creation of the pEAQ series of vectors that were smaller and more simplified (Sainsbury et al., 2009). The study utilized the pEAQ-HT vector to transiently express the four main structural proteins of the bluetongue virus (VP3, VP7, VP5, and VP2) in sheep. The results showed that Virus-like particles (VLPs) are formed, and purified VLPs have been found to induce a defensive immune response in sheep (Thuenemann et al., 2013).

Several viral vector systems utilizing geminiviruses have been reported for In-Plant Activation (INPACT), TYDV-based systems (Dugdale et al., 2013), BeYDV-based systems (Mor et al., 2003; Zhang and Mason, 2006; Huang et al., 2009), which have long/short IR (LIR/SIR) and two replication-related genes (Rep/RepA), and BCTV-based systems (Kim et al., 2007; Chung et al., 2011), which has IR and three replication-related genes (Rep, TrAP and REn). Using these viral vector systems, very high yields of recombinant proteins were produced, including 0.8 mg/g FW HBcAg, 0.34 mg/g FW Norwalk virus coat protein (Huang et al., 2009), 0.5 mg/g FW anti-ebola monoclonal antibody 6D8 (Huang et al., 2010), and 0.55 mg/g FW human papillomavirus coat protein L1 (Regnard et al., 2010).

In this study, up to 1.2 mg/g FW of tGFP was produced (**Figure 5E**), showing a high level of expression. However, tGFP production was relatively low compared to 5 mg/g FW GFP using the magnICON system (Marillonnet et al., 2004) or 1.6 g/kg FW GFP using a CPMV-based viral vector (Sainsbury and Lomonossoff, 2008). This expression was achieved by episomal replicon formation of the *tGFP* expression cassette by geminivirus-based viral vectors, resulting in a dramatically higher number of copies and, correspondingly, higher mRNA and protein expression levels (**Supplementary Figure S1**). The *tGFP* expression cassette formed a circular episomal replicon under the interaction between IRs at both ends and the proteins encoding replication-related genes (**Figures 2A, B**) and had a copy number approximately 700-fold higher than that of tGFP-only expression (**Figures 2C, D**). This is consistent with reports that vectors that replicate have 100-1000-fold higher copy numbers than those that do not replicate (Regnard et al., 2010). Furthermore, at 3 DPI, the *tGFP* transcript level of TIRtGFP+TC123 increased 7.9-fold compared to that of *tGFP* expression alone (**Figure 3A**). However, compared with the dramatic increase in copy number, the increase in transcript levels was only approximately 1.1%. This tendency is consistent with the results of recombinant protein expression in plants with geminivirus-based viral vectors (Huang et al., 2009; Regnard et al., 2010).

In the case of enhancement of transient expression of *tGFP* by the replication-related genes TYLCV and HYVV, the synergistic effect of C123 was the highest, and C12 was not statistically significant compared to C123 (**Figures 3D, E**). However, in the case of BMCTV, it was highest in C1, but C12 and C123 had an antagonistic effect, resulting in a significant decrease in fluorescence compared to tGFP alone (**Figure 3F**). BMCTV also showed little increase in tGFP fluorescence by C1, unlike TYLCV and HYVV, and the overall *tGFP* expression was relatively weak (**Figures 3D–F**). This seemingly correlated with the mild symptoms in BMCTV-infected plants (Soto and Gilbertson, 2003; Strausbaugh et al., 2008).

As shown in **Figure 3**, the expression of *tGFP* using the geminivirus-based vector was highest at 3 DPI, and the infiltrated leaves showed a slightly pale color, but the expression decreased at 5 and 7 DPI. However, the leaves infiltrated with both Rep and tGFP showed a pale green color and leaf curl. This is consistent with reports that when recombinant proteins are expressed using geminivirus-based vectors, protein expression is the highest in the early stage after infiltration, followed by a rapid decrease in protein expression by gene silencing and the subsequent induction of chlorosis and necrosis due to an increase in Rep and Rep-induced replicons (Dugdale et al., 2013; Peyret and Lomonossoff, 2015; Diamos and Mason, 2018a). Therefore, the co-expression of a silencing suppressor, such as p19, has been reported to help increase recombinant protein expression. When the p19 gene-silencing suppressor was added, transient GFP expression by the BCTV-based vector increased 1.6-fold increased (Kim et al., 2007).

When comparing the transcript levels of co-infiltration without p19 and with p19, we observed that the peak of the former was at 1 DPI, whereas that of the latter shifted to 3 DPI. This indicated that p19 suppressed PTGS expression, resulting in increased expression (**Figure 4A**). We also observed that the co-infiltration of p19 resulted in stronger tGFP fluorescence (**Figure 4B**). TC123+TIRtGFP+p19 and HC123+HIRtGFP+p19 peaked at 3 DPI, whereas BC1+BIRtGFP+p19 peaked at 5 DPI. Furthermore, although the fluorescence of BC1+BIRtGFP+p19 at 5 DPI was the highest compared to that of the others, it was only approximately 73–82% of the fluorescence at 3 DPI compared to the other cases (**Figure 4B**). At 3 DPI, TC123+TIRtGFP showed a 7.9-fold increase in transcript levels and a 3.1-fold increase in fluorescence compared with tGFP, whereas TC123+TIRtGFP+P19 showed a 27.7-fold increase in transcript levels and a 7.1-fold increase in fluorescence compared with tGFP (**Figures 3**, **4**). This indicated that p19 co-infiltration increased transcript levels by approximately 3.5-fold and fluorescence by approximately 2.3-fold. Protein electrophoresis and western blotting also showed that tGFP accumulated at much higher levels in the presence of p19 (**Figures 5B, D**).

## 5 Conclusion

Overall, we found that TYLCV- and HYVV-based viral vectors are suitable for high production of recombinant proteins. Although the yield of recombinant proteins was not as high as that of the other two viral vectors, the BMCTV-based viral vector had the potential to produce moderately high yields with less necrosis when expressing animal/virus-derived proteins, given the mild symptoms and an increase in fluorescence at 5 DPI with the addition of p19. We expected that BMCTV-based viral vectors might have an advantage over other viral vectors that offer a higher accumulation of recombinant proteins but have the potential for reduced biomass due to necrosis.

Therefore, we anticipated that these viral vectors could also be used to express viral antigenic or therapeutic proteins of animal/human origin with a high yield of recombinant proteins, and we are currently conducting experiments to prove this. Experimental validation is essential for selecting optimal vector elements to further increase the yield. These elements include a strong promoter such as the cassava vein mosaic virus (CsVMV) promoter (Kim et al., 2007), a 5′-UTR as a strong translation enhancer, a double terminator combination that is known to further increase recombinant protein production and reduce necrosis, and the addition of a matrix attachment region at the 3′-end of the terminator (Diamos and Mason, 2018b).

## Data availability statement

The datasets presented in this study can be found in online repositories. The names of the repository/repositories and accession number(s) can be found in the article/**Supplementary Material**.

## Author contributions

N-SK: Investigation, Methodology, Visualization, Writing – original draft, Writing – review & editing. K-RL: Conceptualization, Funding acquisition, Supervision, Writing – original draft, Writing – review & editing. JiL: Investigation, Methodology, Writing – review & editing. E-JK: Methodology, Writing – review & editing. JuL: Investigation, Methodology, Writing – review & editing. S-KL: Investigation, Methodology, Writing – review & editing.
