# Supplementary material for: High production of recombinant protein using geminivirus-based deconstructed vectors in Nicotiana benthamiana
Source: Front Plant Sci. 2024 Jul 23;15:1407240. doi: 10.3389/fpls.2024.1407240 (PMC11300340; doi:10.3389/fpls.2024.1407240)
Supplement: Supplementary file 1 [file Presentation_1.pptx]

## Slide 1
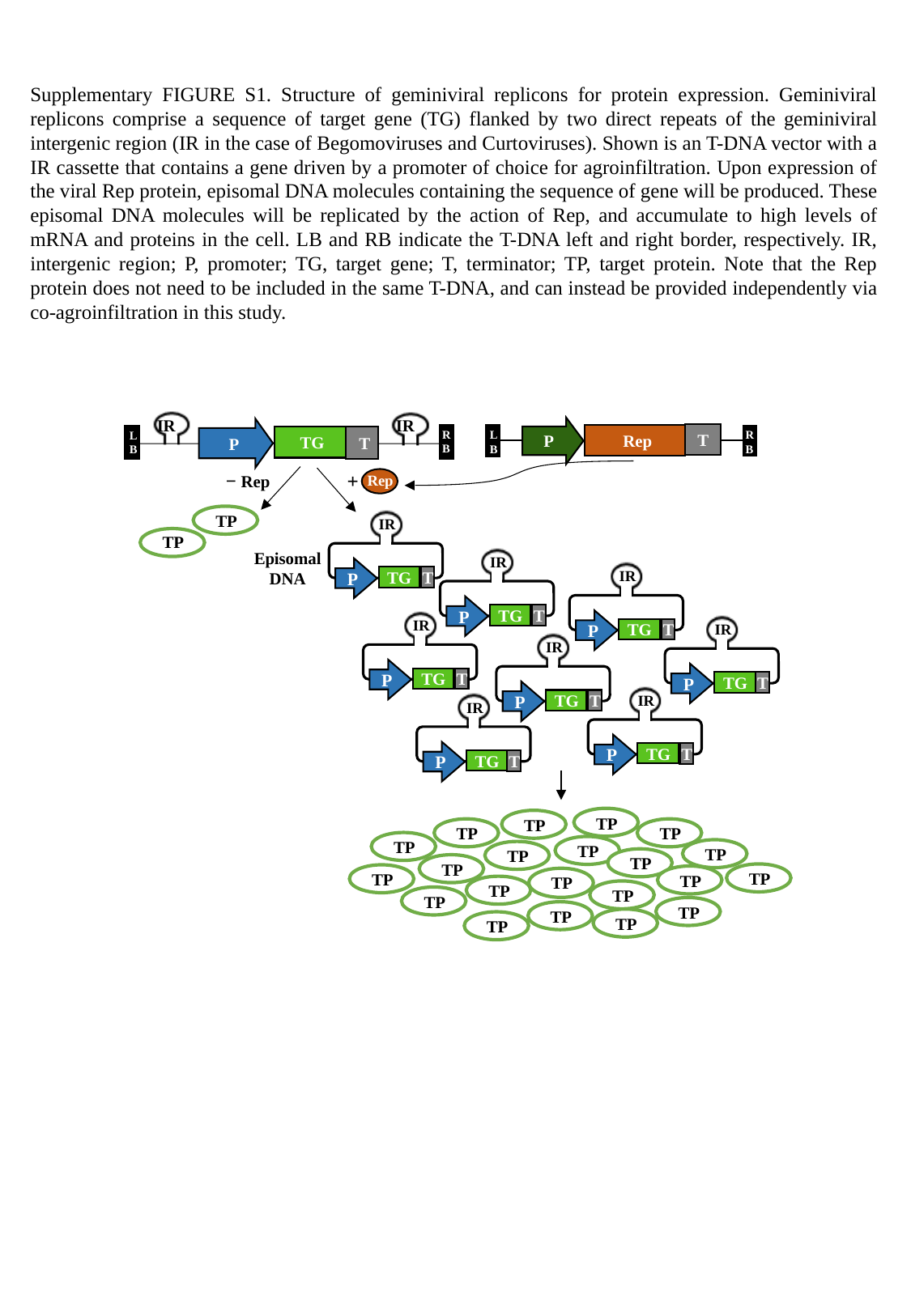

Supplementary FIGURE S1. Structure of geminiviral replicons for protein expression. Geminiviral replicons comprise a sequence of target gene (TG) flanked by two direct repeats of the geminiviral intergenic region (IR in the case of Begomoviruses and Curtoviruses). Shown is an T-DNA vector with a IR cassette that contains a gene driven by a promoter of choice for agroinfiltration. Upon expression of the viral Rep protein, episomal DNA molecules containing the sequence of gene will be produced. These episomal DNA molecules will be replicated by the action of Rep, and accumulate to high levels of mRNA and proteins in the cell. LB and RB indicate the T-DNA left and right border, respectively. IR, intergenic region; P, promoter; TG, target gene; T, terminator; TP, target protein. Note that the Rep protein does not need to be included in the same T-DNA, and can instead be provided independently via co-agroinfiltration in this study.
IR
IR
 P
R
B
L
B
 T
 TG
 P
L
B
R
B
 T
 Rep
+
Rep
− Rep
 TP
 TP
IR
P
 TG
T
Episomal
DNA
IR
P
 TG
T
IR
P
 TG
T
IR
P
 TG
T
IR
P
 TG
T
IR
P
 TG
T
IR
P
 TG
T
IR
P
 TG
T
 TP
 TP
 TP
 TP
 TP
 TP
 TP
 TP
 TP
 TP
 TP
 TP
 TP
 TP
 TP
 TP
 TP
 TP
 TP
 TP
 TP

## Slide 2
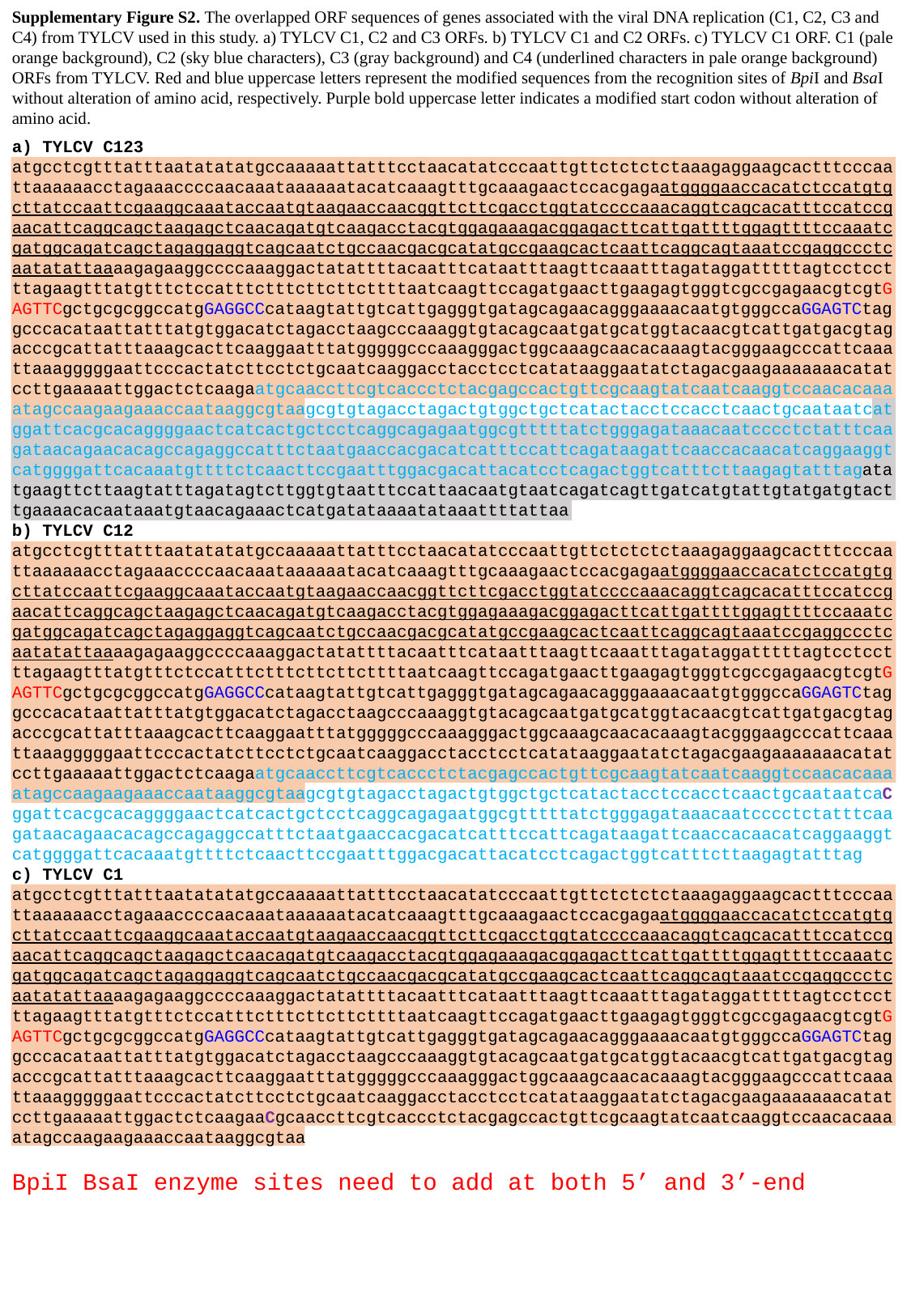

Supplementary Figure S2. The overlapped ORF sequences of genes associated with the viral DNA replication (C1, C2, C3 and C4) from TYLCV used in this study. a) TYLCV C1, C2 and C3 ORFs. b) TYLCV C1 and C2 ORFs. c) TYLCV C1 ORF. C1 (pale orange background), C2 (sky blue characters), C3 (gray background) and C4 (underlined characters in pale orange background) ORFs from TYLCV. Red and blue uppercase letters represent the modified sequences from the recognition sites of BpiI and BsaI without alteration of amino acid, respectively. Purple bold uppercase letter indicates a modified start codon without alteration of amino acid.
a) TYLCV C123
atgcctcgtttatttaatatatatgccaaaaattatttcctaacatatcccaattgttctctctctaaagaggaagcactttcccaattaaaaaacctagaaaccccaacaaataaaaaatacatcaaagtttgcaaagaactccacgagaatggggaaccacatctccatgtgcttatccaattcgaaggcaaataccaatgtaagaaccaacggttcttcgacctggtatccccaaacaggtcagcacatttccatccgaacattcaggcagctaagagctcaacagatgtcaagacctacgtggagaaagacggagacttcattgattttggagttttccaaatcgatggcagatcagctagaggaggtcagcaatctgccaacgacgcatatgccgaagcactcaattcaggcagtaaatccgaggccctcaatatattaaaagagaaggccccaaaggactatattttacaatttcataatttaagttcaaatttagataggatttttagtcctcctttagaagtttatgtttctccatttctttcttcttcttttaatcaagttccagatgaacttgaagagtgggtcgccgagaacgtcgtGAGTTCgctgcgcggccatgGAGGCCcataagtattgtcattgagggtgatagcagaacagggaaaacaatgtgggccaGGAGTCtaggcccacataattatttatgtggacatctagacctaagcccaaaggtgtacagcaatgatgcatggtacaacgtcattgatgacgtagacccgcattatttaaagcacttcaaggaatttatgggggcccaaagggactggcaaagcaacacaaagtacgggaagcccattcaaattaaagggggaattcccactatcttcctctgcaatcaaggacctacctcctcatataaggaatatctagacgaagaaaaaaacatatccttgaaaaattggactctcaagaatgcaaccttcgtcaccctctacgagccactgttcgcaagtatcaatcaaggtccaacacaaaatagccaagaagaaaccaataaggcgtaagcgtgtagacctagactgtggctgctcatactacctccacctcaactgcaataatcatggattcacgcacaggggaactcatcactgctcctcaggcagagaatggcgtttttatctgggagataaacaatcccctctatttcaagataacagaacacagccagaggccatttctaatgaaccacgacatcatttccattcagataagattcaaccacaacatcaggaaggtcatggggattcacaaatgttttctcaacttccgaatttggacgacattacatcctcagactggtcatttcttaagagtatttagatatgaagttcttaagtatttagatagtcttggtgtaatttccattaacaatgtaatcagatcagttgatcatgtattgtatgatgtacttgaaaacacaataaatgtaacagaaactcatgatataaaatataaattttattaa
b) TYLCV C12
atgcctcgtttatttaatatatatgccaaaaattatttcctaacatatcccaattgttctctctctaaagaggaagcactttcccaattaaaaaacctagaaaccccaacaaataaaaaatacatcaaagtttgcaaagaactccacgagaatggggaaccacatctccatgtgcttatccaattcgaaggcaaataccaatgtaagaaccaacggttcttcgacctggtatccccaaacaggtcagcacatttccatccgaacattcaggcagctaagagctcaacagatgtcaagacctacgtggagaaagacggagacttcattgattttggagttttccaaatcgatggcagatcagctagaggaggtcagcaatctgccaacgacgcatatgccgaagcactcaattcaggcagtaaatccgaggccctcaatatattaaaagagaaggccccaaaggactatattttacaatttcataatttaagttcaaatttagataggatttttagtcctcctttagaagtttatgtttctccatttctttcttcttcttttaatcaagttccagatgaacttgaagagtgggtcgccgagaacgtcgtGAGTTCgctgcgcggccatgGAGGCCcataagtattgtcattgagggtgatagcagaacagggaaaacaatgtgggccaGGAGTCtaggcccacataattatttatgtggacatctagacctaagcccaaaggtgtacagcaatgatgcatggtacaacgtcattgatgacgtagacccgcattatttaaagcacttcaaggaatttatgggggcccaaagggactggcaaagcaacacaaagtacgggaagcccattcaaattaaagggggaattcccactatcttcctctgcaatcaaggacctacctcctcatataaggaatatctagacgaagaaaaaaacatatccttgaaaaattggactctcaagaatgcaaccttcgtcaccctctacgagccactgttcgcaagtatcaatcaaggtccaacacaaaatagccaagaagaaaccaataaggcgtaagcgtgtagacctagactgtggctgctcatactacctccacctcaactgcaataatcaCggattcacgcacaggggaactcatcactgctcctcaggcagagaatggcgtttttatctgggagataaacaatcccctctatttcaagataacagaacacagccagaggccatttctaatgaaccacgacatcatttccattcagataagattcaaccacaacatcaggaaggtcatggggattcacaaatgttttctcaacttccgaatttggacgacattacatcctcagactggtcatttcttaagagtatttag
c) TYLCV C1
atgcctcgtttatttaatatatatgccaaaaattatttcctaacatatcccaattgttctctctctaaagaggaagcactttcccaattaaaaaacctagaaaccccaacaaataaaaaatacatcaaagtttgcaaagaactccacgagaatggggaaccacatctccatgtgcttatccaattcgaaggcaaataccaatgtaagaaccaacggttcttcgacctggtatccccaaacaggtcagcacatttccatccgaacattcaggcagctaagagctcaacagatgtcaagacctacgtggagaaagacggagacttcattgattttggagttttccaaatcgatggcagatcagctagaggaggtcagcaatctgccaacgacgcatatgccgaagcactcaattcaggcagtaaatccgaggccctcaatatattaaaagagaaggccccaaaggactatattttacaatttcataatttaagttcaaatttagataggatttttagtcctcctttagaagtttatgtttctccatttctttcttcttcttttaatcaagttccagatgaacttgaagagtgggtcgccgagaacgtcgtGAGTTCgctgcgcggccatgGAGGCCcataagtattgtcattgagggtgatagcagaacagggaaaacaatgtgggccaGGAGTCtaggcccacataattatttatgtggacatctagacctaagcccaaaggtgtacagcaatgatgcatggtacaacgtcattgatgacgtagacccgcattatttaaagcacttcaaggaatttatgggggcccaaagggactggcaaagcaacacaaagtacgggaagcccattcaaattaaagggggaattcccactatcttcctctgcaatcaaggacctacctcctcatataaggaatatctagacgaagaaaaaaacatatccttgaaaaattggactctcaagaaCgcaaccttcgtcaccctctacgagccactgttcgcaagtatcaatcaaggtccaacacaaaatagccaagaagaaaccaataaggcgtaa
BpiI BsaI enzyme sites need to add at both 5’ and 3’-end

## Slide 3
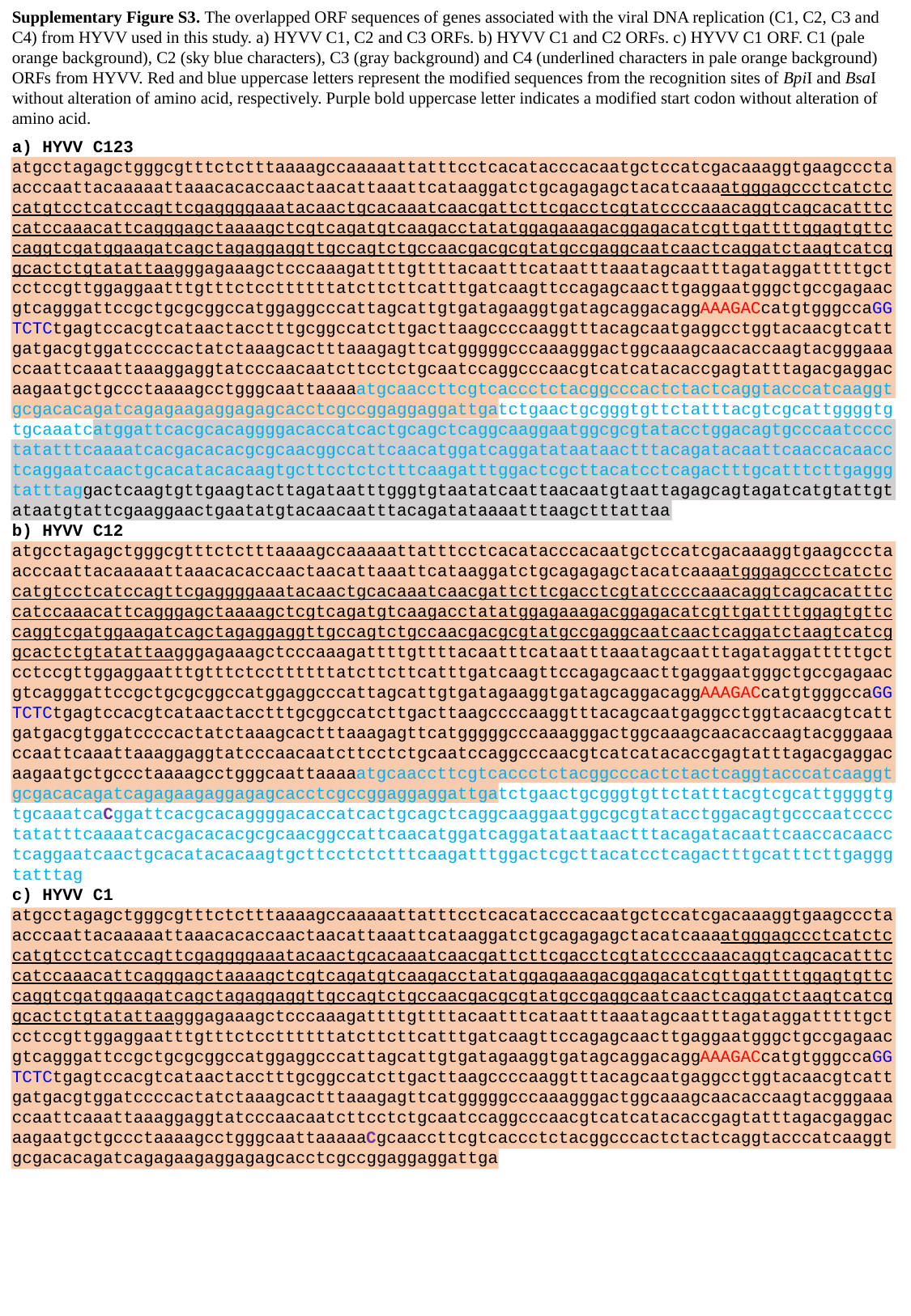

Supplementary Figure S3. The overlapped ORF sequences of genes associated with the viral DNA replication (C1, C2, C3 and C4) from HYVV used in this study. a) HYVV C1, C2 and C3 ORFs. b) HYVV C1 and C2 ORFs. c) HYVV C1 ORF. C1 (pale orange background), C2 (sky blue characters), C3 (gray background) and C4 (underlined characters in pale orange background) ORFs from HYVV. Red and blue uppercase letters represent the modified sequences from the recognition sites of BpiI and BsaI without alteration of amino acid, respectively. Purple bold uppercase letter indicates a modified start codon without alteration of amino acid.
a) HYVV C123
atgcctagagctgggcgtttctctttaaaagccaaaaattatttcctcacatacccacaatgctccatcgacaaaggtgaagccctaacccaattacaaaaattaaacacaccaactaacattaaattcataaggatctgcagagagctacatcaaaatgggagccctcatctccatgtcctcatccagttcgaggggaaatacaactgcacaaatcaacgattcttcgacctcgtatccccaaacaggtcagcacatttccatccaaacattcagggagctaaaagctcgtcagatgtcaagacctatatggagaaagacggagacatcgttgattttggagtgttccaggtcgatggaagatcagctagaggaggttgccagtctgccaacgacgcgtatgccgaggcaatcaactcaggatctaagtcatcggcactctgtatattaagggagaaagctcccaaagattttgttttacaatttcataatttaaatagcaatttagataggatttttgctcctccgttggaggaatttgtttctccttttttatcttcttcatttgatcaagttccagagcaacttgaggaatgggctgccgagaacgtcagggattccgctgcgcggccatggaggcccattagcattgtgatagaaggtgatagcaggacaggAAAGACcatgtgggccaGGTCTCtgagtccacgtcataactacctttgcggccatcttgacttaagccccaaggtttacagcaatgaggcctggtacaacgtcattgatgacgtggatccccactatctaaagcactttaaagagttcatgggggcccaaagggactggcaaagcaacaccaagtacgggaaaccaattcaaattaaaggaggtatcccaacaatcttcctctgcaatccaggcccaacgtcatcatacaccgagtatttagacgaggacaagaatgctgccctaaaagcctgggcaattaaaaatgcaaccttcgtcaccctctacggcccactctactcaggtacccatcaaggtgcgacacagatcagagaagaggagagcacctcgccggaggaggattgatctgaactgcgggtgttctatttacgtcgcattggggtgtgcaaatcatggattcacgcacaggggacaccatcactgcagctcaggcaaggaatggcgcgtatacctggacagtgcccaatcccctatatttcaaaatcacgacacacgcgcaacggccattcaacatggatcaggatataataactttacagatacaattcaaccacaacctcaggaatcaactgcacatacacaagtgcttcctctctttcaagatttggactcgcttacatcctcagactttgcatttcttgagggtatttaggactcaagtgttgaagtacttagataatttgggtgtaatatcaattaacaatgtaattagagcagtagatcatgtattgtataatgtattcgaaggaactgaatatgtacaacaatttacagatataaaatttaagctttattaa
b) HYVV C12
atgcctagagctgggcgtttctctttaaaagccaaaaattatttcctcacatacccacaatgctccatcgacaaaggtgaagccctaacccaattacaaaaattaaacacaccaactaacattaaattcataaggatctgcagagagctacatcaaaatgggagccctcatctccatgtcctcatccagttcgaggggaaatacaactgcacaaatcaacgattcttcgacctcgtatccccaaacaggtcagcacatttccatccaaacattcagggagctaaaagctcgtcagatgtcaagacctatatggagaaagacggagacatcgttgattttggagtgttccaggtcgatggaagatcagctagaggaggttgccagtctgccaacgacgcgtatgccgaggcaatcaactcaggatctaagtcatcggcactctgtatattaagggagaaagctcccaaagattttgttttacaatttcataatttaaatagcaatttagataggatttttgctcctccgttggaggaatttgtttctccttttttatcttcttcatttgatcaagttccagagcaacttgaggaatgggctgccgagaacgtcagggattccgctgcgcggccatggaggcccattagcattgtgatagaaggtgatagcaggacaggAAAGACcatgtgggccaGGTCTCtgagtccacgtcataactacctttgcggccatcttgacttaagccccaaggtttacagcaatgaggcctggtacaacgtcattgatgacgtggatccccactatctaaagcactttaaagagttcatgggggcccaaagggactggcaaagcaacaccaagtacgggaaaccaattcaaattaaaggaggtatcccaacaatcttcctctgcaatccaggcccaacgtcatcatacaccgagtatttagacgaggacaagaatgctgccctaaaagcctgggcaattaaaaatgcaaccttcgtcaccctctacggcccactctactcaggtacccatcaaggtgcgacacagatcagagaagaggagagcacctcgccggaggaggattgatctgaactgcgggtgttctatttacgtcgcattggggtgtgcaaatcaCggattcacgcacaggggacaccatcactgcagctcaggcaaggaatggcgcgtatacctggacagtgcccaatcccctatatttcaaaatcacgacacacgcgcaacggccattcaacatggatcaggatataataactttacagatacaattcaaccacaacctcaggaatcaactgcacatacacaagtgcttcctctctttcaagatttggactcgcttacatcctcagactttgcatttcttgagggtatttag
c) HYVV C1
atgcctagagctgggcgtttctctttaaaagccaaaaattatttcctcacatacccacaatgctccatcgacaaaggtgaagccctaacccaattacaaaaattaaacacaccaactaacattaaattcataaggatctgcagagagctacatcaaaatgggagccctcatctccatgtcctcatccagttcgaggggaaatacaactgcacaaatcaacgattcttcgacctcgtatccccaaacaggtcagcacatttccatccaaacattcagggagctaaaagctcgtcagatgtcaagacctatatggagaaagacggagacatcgttgattttggagtgttccaggtcgatggaagatcagctagaggaggttgccagtctgccaacgacgcgtatgccgaggcaatcaactcaggatctaagtcatcggcactctgtatattaagggagaaagctcccaaagattttgttttacaatttcataatttaaatagcaatttagataggatttttgctcctccgttggaggaatttgtttctccttttttatcttcttcatttgatcaagttccagagcaacttgaggaatgggctgccgagaacgtcagggattccgctgcgcggccatggaggcccattagcattgtgatagaaggtgatagcaggacaggAAAGACcatgtgggccaGGTCTCtgagtccacgtcataactacctttgcggccatcttgacttaagccccaaggtttacagcaatgaggcctggtacaacgtcattgatgacgtggatccccactatctaaagcactttaaagagttcatgggggcccaaagggactggcaaagcaacaccaagtacgggaaaccaattcaaattaaaggaggtatcccaacaatcttcctctgcaatccaggcccaacgtcatcatacaccgagtatttagacgaggacaagaatgctgccctaaaagcctgggcaattaaaaaCgcaaccttcgtcaccctctacggcccactctactcaggtacccatcaaggtgcgacacagatcagagaagaggagagcacctcgccggaggaggattga

## Slide 4
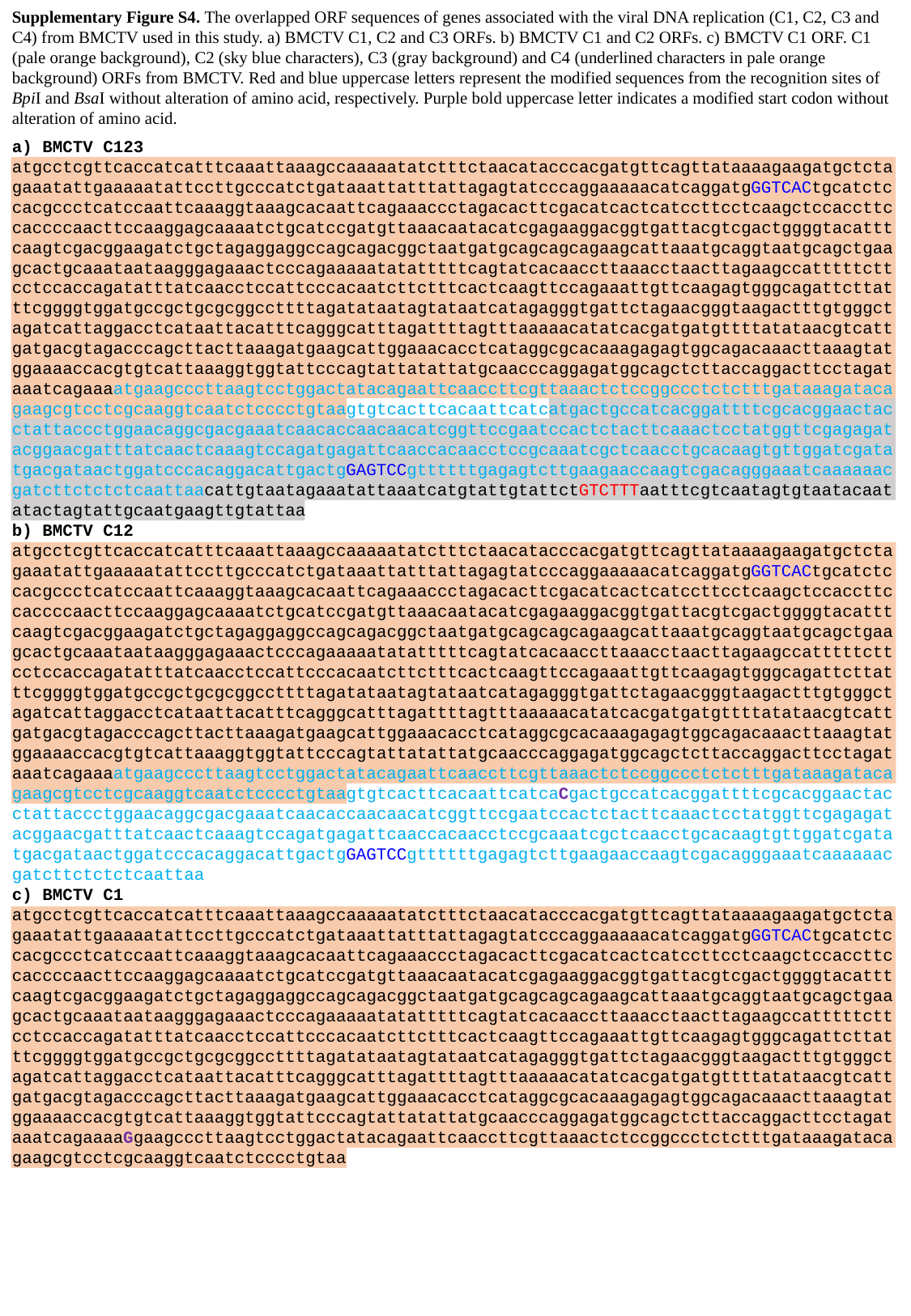

Supplementary Figure S4. The overlapped ORF sequences of genes associated with the viral DNA replication (C1, C2, C3 and C4) from BMCTV used in this study. a) BMCTV C1, C2 and C3 ORFs. b) BMCTV C1 and C2 ORFs. c) BMCTV C1 ORF. C1 (pale orange background), C2 (sky blue characters), C3 (gray background) and C4 (underlined characters in pale orange background) ORFs from BMCTV. Red and blue uppercase letters represent the modified sequences from the recognition sites of BpiI and BsaI without alteration of amino acid, respectively. Purple bold uppercase letter indicates a modified start codon without alteration of amino acid.
a) BMCTV C123
atgcctcgttcaccatcatttcaaattaaagccaaaaatatctttctaacatacccacgatgttcagttataaaagaagatgctctagaaatattgaaaaatattccttgcccatctgataaattatttattagagtatcccaggaaaaacatcaggatgGGTCACtgcatctccacgccctcatccaattcaaaggtaaagcacaattcagaaaccctagacacttcgacatcactcatccttcctcaagctccaccttccaccccaacttccaaggagcaaaatctgcatccgatgttaaacaatacatcgagaaggacggtgattacgtcgactggggtacatttcaagtcgacggaagatctgctagaggaggccagcagacggctaatgatgcagcagcagaagcattaaatgcaggtaatgcagctgaagcactgcaaataataagggagaaactcccagaaaaatatatttttcagtatcacaaccttaaacctaacttagaagccatttttcttcctccaccagatatttatcaacctccattcccacaatcttctttcactcaagttccagaaattgttcaagagtgggcagattcttatttcggggtggatgccgctgcgcggccttttagatataatagtataatcatagagggtgattctagaacgggtaagactttgtgggctagatcattaggacctcataattacatttcagggcatttagattttagtttaaaaacatatcacgatgatgttttatataacgtcattgatgacgtagacccagcttacttaaagatgaagcattggaaacacctcataggcgcacaaagagagtggcagacaaacttaaagtatggaaaaccacgtgtcattaaaggtggtattcccagtattatattatgcaacccaggagatggcagctcttaccaggacttcctagataaatcagaaaatgaagcccttaagtcctggactatacagaattcaaccttcgttaaactctccggccctctctttgataaagatacagaagcgtcctcgcaaggtcaatctcccctgtaagtgtcacttcacaattcatcatgactgccatcacggattttcgcacggaactacctattaccctggaacaggcgacgaaatcaacaccaacaacatcggttccgaatccactctacttcaaactcctatggttcgagagatacggaacgatttatcaactcaaagtccagatgagattcaaccacaacctccgcaaatcgctcaacctgcacaagtgttggatcgatatgacgataactggatcccacaggacattgactgGAGTCCgttttttgagagtcttgaagaaccaagtcgacagggaaatcaaaaaacgatcttctctctcaattaacattgtaatagaaatattaaatcatgtattgtattctGTCTTTaatttcgtcaatagtgtaatacaatatactagtattgcaatgaagttgtattaa
b) BMCTV C12
atgcctcgttcaccatcatttcaaattaaagccaaaaatatctttctaacatacccacgatgttcagttataaaagaagatgctctagaaatattgaaaaatattccttgcccatctgataaattatttattagagtatcccaggaaaaacatcaggatgGGTCACtgcatctccacgccctcatccaattcaaaggtaaagcacaattcagaaaccctagacacttcgacatcactcatccttcctcaagctccaccttccaccccaacttccaaggagcaaaatctgcatccgatgttaaacaatacatcgagaaggacggtgattacgtcgactggggtacatttcaagtcgacggaagatctgctagaggaggccagcagacggctaatgatgcagcagcagaagcattaaatgcaggtaatgcagctgaagcactgcaaataataagggagaaactcccagaaaaatatatttttcagtatcacaaccttaaacctaacttagaagccatttttcttcctccaccagatatttatcaacctccattcccacaatcttctttcactcaagttccagaaattgttcaagagtgggcagattcttatttcggggtggatgccgctgcgcggccttttagatataatagtataatcatagagggtgattctagaacgggtaagactttgtgggctagatcattaggacctcataattacatttcagggcatttagattttagtttaaaaacatatcacgatgatgttttatataacgtcattgatgacgtagacccagcttacttaaagatgaagcattggaaacacctcataggcgcacaaagagagtggcagacaaacttaaagtatggaaaaccacgtgtcattaaaggtggtattcccagtattatattatgcaacccaggagatggcagctcttaccaggacttcctagataaatcagaaaatgaagcccttaagtcctggactatacagaattcaaccttcgttaaactctccggccctctctttgataaagatacagaagcgtcctcgcaaggtcaatctcccctgtaagtgtcacttcacaattcatcaCgactgccatcacggattttcgcacggaactacctattaccctggaacaggcgacgaaatcaacaccaacaacatcggttccgaatccactctacttcaaactcctatggttcgagagatacggaacgatttatcaactcaaagtccagatgagattcaaccacaacctccgcaaatcgctcaacctgcacaagtgttggatcgatatgacgataactggatcccacaggacattgactgGAGTCCgttttttgagagtcttgaagaaccaagtcgacagggaaatcaaaaaacgatcttctctctcaattaa
c) BMCTV C1
atgcctcgttcaccatcatttcaaattaaagccaaaaatatctttctaacatacccacgatgttcagttataaaagaagatgctctagaaatattgaaaaatattccttgcccatctgataaattatttattagagtatcccaggaaaaacatcaggatgGGTCACtgcatctccacgccctcatccaattcaaaggtaaagcacaattcagaaaccctagacacttcgacatcactcatccttcctcaagctccaccttccaccccaacttccaaggagcaaaatctgcatccgatgttaaacaatacatcgagaaggacggtgattacgtcgactggggtacatttcaagtcgacggaagatctgctagaggaggccagcagacggctaatgatgcagcagcagaagcattaaatgcaggtaatgcagctgaagcactgcaaataataagggagaaactcccagaaaaatatatttttcagtatcacaaccttaaacctaacttagaagccatttttcttcctccaccagatatttatcaacctccattcccacaatcttctttcactcaagttccagaaattgttcaagagtgggcagattcttatttcggggtggatgccgctgcgcggccttttagatataatagtataatcatagagggtgattctagaacgggtaagactttgtgggctagatcattaggacctcataattacatttcagggcatttagattttagtttaaaaacatatcacgatgatgttttatataacgtcattgatgacgtagacccagcttacttaaagatgaagcattggaaacacctcataggcgcacaaagagagtggcagacaaacttaaagtatggaaaaccacgtgtcattaaaggtggtattcccagtattatattatgcaacccaggagatggcagctcttaccaggacttcctagataaatcagaaaaGgaagcccttaagtcctggactatacagaattcaaccttcgttaaactctccggccctctctttgataaagatacagaagcgtcctcgcaaggtcaatctcccctgtaa

## Slide 5
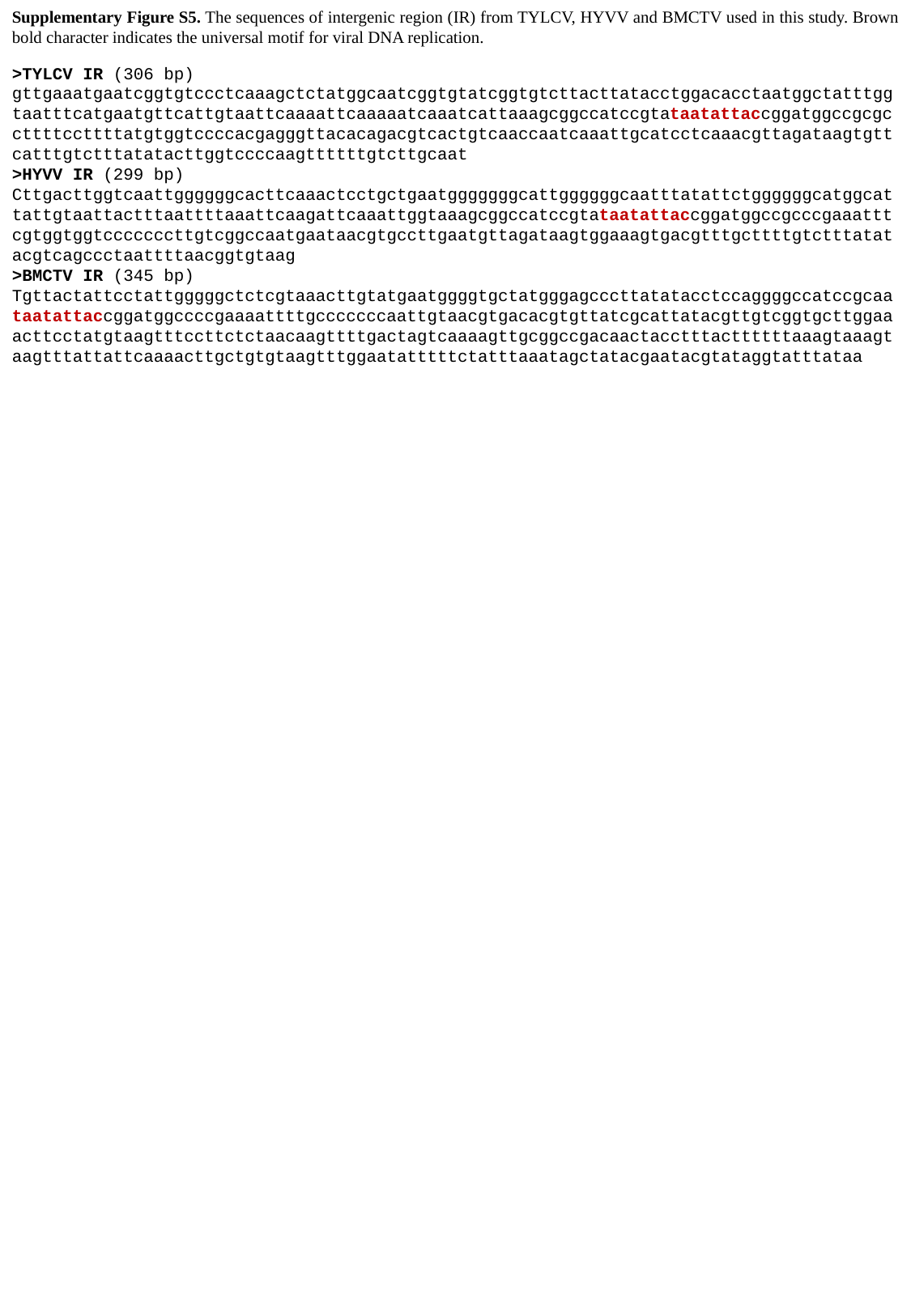

Supplementary Figure S5. The sequences of intergenic region (IR) from TYLCV, HYVV and BMCTV used in this study. Brown bold character indicates the universal motif for viral DNA replication.
>TYLCV IR (306 bp)
gttgaaatgaatcggtgtccctcaaagctctatggcaatcggtgtatcggtgtcttacttatacctggacacctaatggctatttggtaatttcatgaatgttcattgtaattcaaaattcaaaaatcaaatcattaaagcggccatccgtataatattaccggatggccgcgccttttccttttatgtggtccccacgagggttacacagacgtcactgtcaaccaatcaaattgcatcctcaaacgttagataagtgttcatttgtctttatatacttggtccccaagttttttgtcttgcaat
>HYVV IR (299 bp)
Cttgacttggtcaattggggggcacttcaaactcctgctgaatgggggggcattggggggcaatttatattctggggggcatggcattattgtaattactttaattttaaattcaagattcaaattggtaaagcggccatccgtataatattaccggatggccgcccgaaatttcgtggtggtcccccccttgtcggccaatgaataacgtgccttgaatgttagataagtggaaagtgacgtttgcttttgtctttatatacgtcagccctaattttaacggtgtaag
>BMCTV IR (345 bp)
Tgttactattcctattgggggctctcgtaaacttgtatgaatggggtgctatgggagcccttatatacctccaggggccatccgcaataatattaccggatggccccgaaaattttgcccccccaattgtaacgtgacacgtgttatcgcattatacgttgtcggtgcttggaaacttcctatgtaagtttccttctctaacaagttttgactagtcaaaagttgcggccgacaactacctttacttttttaaagtaaagtaagtttattattcaaaacttgctgtgtaagtttggaatatttttctatttaaatagctatacgaatacgtataggtatttataa
