# Supplementary material for: High production of recombinant protein using geminivirus-based deconstructed vectors in Nicotiana benthamiana
Source: Front Plant Sci. 2024 Jul 23;15:1407240. doi: 10.3389/fpls.2024.1407240 (PMC11300340; doi:10.3389/fpls.2024.1407240)
Supplement: Supplementary file 2 [file Table_1.docx]

| Primer name | DNA sequence (5´-3´) | Purpose |
| --- | --- | --- |
| TYLCV IR F  TYLCV IR R  HYVV IR F  HYVV IR R  BMCTV IR F  BMCTV IR R | TTGAAGACATCTCAGGAGTTGAAATGAATCGGTGTCCCT  TTGAAGACAACTCGAGTATTGCAAGACAAAAAACTTGGG  TTGAAGACATCTCAGGAGTTGACTTGGTCAATTGGG  TTGAAGACAACTCGAGTACTTACACCGTTAAAATTAGGGCTGA  TTGAAGACATCTCAGGAGGTTACTATTCCTATTGGGGGCTCT  TTGAAGACAACTCGAGTATTATAAATACCTATACGTATTCGTATAGC | IR cloning |
| Replicon PT F1  Replicon d35S R2 | TGTGGCCTTAATTGAATCATC  AGAGGAAGGGTCTTGCGAAG | Episomal replicon DNA PCR |
| β-actin F1  β-actin R1  tGFP F1  tGFP R1 | CCACCGGTATTGTGTTGGAC  CCTGACAATTTCCCGCTCAG  AGTTCGAGCTTGTTGGAGGT  TGGGTAAGTTCCGAAGTGGT | qRT-PCR |

Supplementary Table S1. List of primers used in this study.
